# How Shall the Stomatologist Be Educated?*Read in the Section on Stomatology of the American Medical Association, at the Sixty-first Annual Session, at St. Louis, June. 1910.

**Published:** 1910-07-15

**Authors:** M. H. Cryer

**Affiliations:** Professor of Oral Surgery, University of Pennsylvania. Philadelphia


### HOW SHALL THE STOMATOLOGIST BE EDUCATED?*

M. H. CRYER, M.D., D.D.S.
PROFESSOR OF ORAL SURGERY, UNIVERSITY OF PENNSYLVANIA.
PHILADELPHIA.

The question of medical education for the dentist has been under discussion for many years, and it will probably continue to be a subject of discussion for many years to come. At the first meeting of the International Association of Stomatology, held in Paris, August, 1907, that body settled to its own satisfaction these two points, first, that a complete medical education must be required of every dentist; second, that the word stomatology was defined as meaning diseases pertaining to the mouth. A stomatologist is one who treats diseases of the mouth. In consideration of these two settled principles, the following series of questions was formulated and discussion requested:

*Read in the Section on Stomatology of the American Medical Association, at the Sixty-first Annual Session, at St. Louis, June, 1910.

(*a*) "When is it best to begin the special studies; during the time of the general medical studies or immediately after these?"

(*b*) "Advantage and disadvantage of each."

(1) "Special studies begun during the general medical course."

(2) "Special studies begun immediately after the general medical course."

(*c*) "Length of time required for special studies of each system."

(*d*) "Details of special studies of each system."

As the words dentist and stomatologist, whether intentionally or not, are accepted in the statements as meaning the same, they will be used synonymously by the writer.

If the first settled statement be correct, that "A complete medical education must be required of every dentist," it demands that, in order to become a practitioner of dentistry o: stomatology, a man must have a partial or full college education, depending on the medical school he enters, must attend a four year medical course, and according to some authorities he must serve as intern in some good general hospital for one and a half to two and a half years. This constitutes a "complete medical education" according to our leading medical authorities. It is very doubtful if such an education should be required of a dentist. But suppose only the four year course in a medical school be demanded; then the student in this country will have to spend three additional years in a dental school, unless he be allowed one year for his medical education. The best dental schools are advocating four years for the dental education; if this should take place then the student would have another year before he would be allowed to take his examination for a license to practice dentistry. According to this ruling a student must devote six or seven years to his professional studies after he leaves college to qualify for practice. By combining or overlapping the work of the two courses, the time spent can be reduced to six and occasionally five years when the two schools of medicine and dentistry are in the same university.

### BEGINNING SPECIAL STUDIES.

With these remarks the writer will endeavor to answer (*a*) and will assume that the two schools belong to the same university. If a student wishes to obtain the two degrees in less than seven years, he so announces it to the deans of both schools and arrangements are made accordingly. At the institution with which I am connected, for the past fifteen years the majority of students combining the two courses have entered both dental and medical schools, and the first year have had the same instruction as those studying medicine alone, viz., the fundamental branches of medical science such as chemistry, anatomy, physiology, and pathology, together with some dental technics. In the second year a similar plan is followed, with an increase of dental work, and if a student has the ability he can complete the first two years of both courses, though he will not be up to the highest standard of those taking single courses in either school. The third year is devoted exclusively to completing his dental course, and he receives the degree D.D.S. at the end of it.

(*b*) Observe now what happens. It is an important point in the discussion, constituting a great objection to a complete medical training, under the present conditions, for a dentist.

1) The last two years of the student's time are spent entirely in the medical school and are occupied chiefly in clinical work. He has not time for anything outside of this. He is thus drawn away from dentistry for at least two years. Not only does he suffer from failure to keep up with new dental methods, but his attention is riveted on the wide field of general medicine. All his surroundings are medical. Even in leisure and social hours he is surrounded by medical associates, joins medical societies, etc. By the time he graduates he finds his classmates taking intern service in leading hospitals, and he naturally desires to do the same. The student drops his dentistry with his D.D.S. and becomes a medical man. Case after case can be cited in which this has happened and but few can be mentioned during the last ten years in which a student who has obtained his dental and medical degrees in the institution spoken of, has practiced dentistry or has made a specialty of oral surgery.

Thirty-six years ago the M.D. and the D.D.S. degrees could be obtained from our leading schools in two years and a half. As the course of instruction in the medical and dental schools lengthened, naturally it required longer time to obtain each degree. Fifteen years ago, and even later, a graduate in dentistry from a reputable school was allowed one year on his medical course. But times have changed; a dental graduate to-day must spend four years in the medical school even if he be a graduate of the same institution, provided he has not combined his studies and taken his work as before mentioned, with that previous understanding.

With this experience, I cannot advocate the plan of taking up dentistry first and finishing with medicine, or of combining the two full courses in any way.

### AFTER MEDICAL COURSE.

(2) The second question to be considered is "Shall the student begin immediately after the general medical course?" If a student has a college education and has taken a four-year-course in a first-class medical school, it is doubtful if he can afford the time or will have the inclination to spend two or three years in a dental school, as his M.D. will allow him to practice any specialty of medicine which includes the diseases of the mouth. However, from experience, the writer does not believe that this finished (?) product would be best equipped to undertake the work of the stomatologist.

(*c*) Length of time required for special studies of each system. It is difficult to answer these questions which are based on the assumption that "A complete medical education must be required of every dentist." as I am not in favor of this plan as settled by the A. S. I.

In my opinion it is almost useless to discuss this subject unless we can get the cooperation of the medical and dental schools. The medical schools of this country have settled, for the present, on certain definite rules by which they give a complete medical education, and are not likely to change their methods for the few dental students who wish a complete medical education. With the exception of the fundamental branches of chemistry, anatomy, physiology, pathology and bacteriology the teaching of the two schools is quite different, and cannot be given by the same teachers.

### SUBJECTS MUST BE SEPARATED.

In many conversations with teachers of medicine and dentistry, I find it generally considered that a student should not undertake these studies jointly. If he is to study medicine he must do so with all his faculties concentrated in that direction. If he is to study dentistry his mind during his study hours must be occupied solely with dentistry; otherwise he will be second to other students who devote their full time to one subject.

If a man wishes to become a specialist in any branch of medicine, whether it be surgery, gynecology or ophthalmology, it is admitted by all medical teachers that that student must take the general course of instruction first. Then after graduation if he decides to become a specialist he can take special work in the branch he selects. In other words, he must be a doctor first. Now, if stomatology is to become a branch of medicine and require the M.D. degree, the student under existing conditions should enter the best medical college possible and take its full teaching. After graduation if he has not changed his mind, he must take up the special work of the dental profession, which will require at least two years, and for most students it should be three; for there is a very large amount of special work connected with dentistry and stomatology.

The plan of the A. S. I., proposing complete medical education of the stomatologist, can only be carried out by leaving the mechanical work of the dentist to persons trained especially for this work. For stomatology to become a specialty of medicine it would have to be given a place in the regular curriculum of medical schools as all the other recognized specialties are, instead of being left to a separate school of dentistry as at the present time; this would mean a complete revolution in dentistry. The training of the stomatologist would extend his field so much that he would be unable to give time to prosthetic or operative work. He would have full charge of medical and surgical cases affecting the mouth and jaws; not only diseases of the teeth and mouth itself, but constitutional diseases having manifestations in the mouth, such as syphilis and gout, also deformities such as harelip and cleft-palate, malignant growths of the jaws and tongue, injuries of the jaws, diseases of the maxillary sinus and associated pneumatic spaces.

Such work as the filling of teeth and dental prosthesis and the making of regulating appliances would be turned over under the stomatologist's direction, or subject to his prescription, to a man specially trained for each division of this work. His relation to this mechanical associate would be analogous to that of the oculist to the optician, the orthopedic surgeon to the maker of braces, the general practitioner to the druggist.

This idea is being carried out even at the present time to a certain extent by leading dentists proportionately as they extend their own fields. Many men have their extraction, prosthetic and regulating work, etc., done by associates under their direction, although in most cases the stomatologist still attends to the filling of teeth; but even this would be dropped if the idea of stomatology as a specialty of medicine were carried out completely.

### SUMMARY.

My views summed up as follows:

The primary object in the study of stomatology or any other profession is not the obtaining of this or that degree, but of gaining that special knowledge which will best equip the student for the practice of his profession. To illustrate this point, many examples may be cited of men of high standard in the dental profession who have the M.D. degree, and also those who have not Both have done and are doing great work for the advancement of dentistry, not because they have this or that degree, but because they have knowledge and the ability to impart it to others and to use it in practice for the good of humanity.

The full medical course is not at present designed to educate students thoroughly in any specialty of medicine. It is impossible to give the student more than a speaking acquaintance with the specialties.

To properly train a student for the practice of stomatology, a special curriculum should be devised consisting of the essential features of both the medical and dental curricula, with the non-essential subjects of both eliminated.

*a.* To begin with, the student must have a good general education and should have considerable mechanical inclination. If he has not the latter he will never make a dentist or stomatologist.

*b.* A period of at least two years should be devoted to the fundamental branches of stomatology comprising chemistry, anatomy, physiology and bacteriology essentially the same as the first two years of a medical course, and in addition, instruction in dental technics and other matters leading to the next period.

*c.* A period of at least two years devoted entirely to the study of the mouth including all branches of clinical dentistry and oral surgery. The latter would comprise all diseases relating to teeth, jaws and associated parts. In this course there would be besides the lectures on oral surgery, ward classes and clinics in a general hospital. At the end of his time the student would receive a degree denominating qualifications to practice stomatology.

*d.* If the student desires to become an oral surgeon he should then have at least one year as resident stomatologist in a large general hospital, affording practical experience not only in operative and prosthetic dentistry, but also in treatment in diseases of the mouth and jaws, such as tumors, necrosis caused by syphilis, phosphorus and tuberculosis, cleft palate and harelip, fractures, diseases of the maxillary sinuses and associated pneumatic spaces.

With such a course as has been outlined the graduate in stomatology would be fully qualified to practice his specialty and would have sufficient knowledge of the fundamental principles of medicine to recognize and discuss intelligently with medical men the relation of diseases of the oral cavity to general pathological conditions and *vice versa.* This plan as regards oral surgical work has been carried out to a large extent for the past nine years by the combined dental schools of Philadelphia in connection with the Philadelphia General Hospital. This hospital has an oral surgical staff consisting of visiting surgeons from the various dental schools and two graduates in dentistry as internes. A clinic is given once a week by a visiting member of the staff, cases of interest to stomatologists are shown and operations on the face and jaw are performed. The clinic is open to graduates and students of medicine and dentistry, many of whom come from all parts of the world, and has the largest attendance of any clinic in Philadelphia. The internes serve from twelve to eighteen months each and have full charge of all cases in which the face, mouth and jaws are involved. They gain experience not only in their specialty, but by coming daily in contact with medical men and studying general pathologic conditions, their views are broadened and they become ideal members of the profession of stomatology.—*Journal A. M. A.*, June 25, 1910.

### ABSTRACT OF DISCUSSION ON PAPERS OF DRS. TALBOT, POWER AND CRYER.

Dr. J. N. Farrar, New York: A complete medical college course of instruction is necessary to success in any branch of medicine. The time needed for such education is the incubating period preparatory to any specialty. No specialty should be attempted without a complete course of medical instruction. Especially is this true in stomatology, if the deeper physiologic lines of the science and art of highest orthodontia are to be well understood.

Dr. Arthur R. Dray, Philadelphia: There was a time when the medical man was required to gather herbs, etc., and to make his own medicines. To-day, with a few exceptions, he has neither time nor qualification to do the work of the pharmacist and druggist. So also to-day we find the dentist, compelled by the many calls on his time, to give much of his work to the mechanic. To-morrow the dentist will cease altogether to splash about with plaster of Paris and flasks, to burn his fingers pouring zinc dies or to smash his thumbs swaging gold plates in an effort to satisfy the patient's needs. The strictly mechanical man, not needing any medical knowledge whatever, will satisfy all these wants, leaving the dentist free to treat diseases and conditions. What does this mean? Why, that the dental student of to-day who spends three or five hours out of every ten in the laboratory, could put this time at the disposal of medical studies and operative work. In the best dental colleges of the United States of to-day ten to fifteen hours or more a week of the schedule periods of the student's roster are spent in the mechanical laboratory learning a trade— a trade that could be better followed by a man devoting his whole time to it, who never need see a patient or operating chair. If in the medical curriculum of any reputable medical college the strictly dental specialties are inserted in place of the strictly medical specialties we have a good working idea of the studies which the dental student would have to take to qualify himself as a dental specialist of medicine. By strictly medical subjects I mean such subjects as orthopedics, gynecology, ophthalmology, obstetrics, mental diseases, medical jurisprudence, pediatrics, etc. The dental subjects should be taught in conjunction with the medical course, being focused on with special effort as the final years are reached.

Four years is not too long a period to spend in preparation for a lifelong work. Five or more years would be a better term. By suggesting the curtailment of the time spent on the strictly medical subjects I do not for one moment wish to imply that they should be altogether slighted, for my belief is and always will be that dentistry is a specialty of medicine and should not be followed until a medical qualification has been received. Let our dental schools of to-day give a special course if they wish, granting a special license and a special degree in mechanical dentistry, this course confining itself entirely to mechanics, lasting but two years; but let them not make an effort to work against impossibilities by teaching medicine and mechanics to the same student in less time than it takes to get a medical degree.

Dr. Eugene H. Smith, Boston, Dean of Harvard University Dental School: To my mind there are but two practical methods of educating young men for dentistry. One method is so to shape their education from the beginning that it will lead directly to the specialty of dentistry; the other is first to give them a general medical education leading to the degree in medicine, and to follow this with two years of special training. In either case the preliminary training should not be less han that obtained in a four years' graded high school, which fits students to pass the entrance requirements to our leading colleges.

By the first method, if we are training students for a dental degree alone, the first year should be devoted to the fundamentals of medicine, namely: histology, physiology, bacteriology, and physiologic and dental chemistry. General chemistry should be required for entrance. The first year medical studies should be planned to give special and advanced training on subjects that especially concern the dentist. The second and third years should be spent in a dental hospital. The clinical work should be carried on in conjunction with lectures, and the students should be taught to make application of the medical work of their first year to the cases they treat in the hospital. I think it a mistake for a dental student to have training in special dental work during his first year of study.

The second method of training a student in dentistry is to enter him as a regular student in a first-class medical school and have him obtain the M.D. degree, after which he should enter on a two years' course in a dental school leading to a dental degree.

Modern dentistry has become so extensive a profession in itself, and modern medicine so vastly different from the medicine of a few years ago, that it is a serious question in my mind whether a student can be properly educated in medicine and in dentistry in less than six full years. If I could be sure that the combined medical and dental training that I have outlined would produce skilled men as well as scientific men I should be strongly in favor of such training. I think, however, that there should be two degrees indicating this training, rather than to have dentistry practiced under the one degree of medicine. We must never lose sight of the fact that in a specialty in which so much technical skill is required it will always be necessary so to shape our courses as to produce skilled practitioners, even though we may do it at an expense of scientific men, using the term "scientific" in its broad sense.

Dr. M. H. Fletcher, Cincinnati: I am in thorough accord with the idea that many dentists, if not all, should have a complete medical education in addition to their mechanical training, since it fits one most satisfactorily for the practice of stomatology. In fact, I do not see how one can be fitted for one practice of stomatology without a full medical education, any more than one can be prepared as an oculist or throat and nose specialist without medical training. No dental college of which I have any knowledge so fits a student, since the dental courses in anatomy, physiology and pathology are decidedly inadequate. The average dental operation can be well performed without much knowledge of the laws of health and disease of the tissues, but the successful treatment of diseased tissues in the mouth and their systemic effects requires a knowledge such as can be attained only by taking a complete course in medicine and surgery.

My own judgment is that we should have stomatologists and dentists; let the dentists stop where they do now, but a student in stomatology should first take his work in the mechanics of dentistry and follow this by special medical work, the two overlapping in the middle of the course, for instance the mechanical overlapping the medical in the later part of the second year or the beginning of the third, ending the course with the medical work in the fourth or fifth year according to the length of the course. In a five-year course (which I think is short enough) the first two years should be devoted to dentistry, the medical studies beginning in the third year while the dental work is being finished. The last two years should be devoted to a medical course and hospital work such as is now required of medical graduates in the average American medical college with clinics and practice in higher dentistry, oral surgery and treatment of diseases of the mouth.

The difficulty about the practical working of this or any plan requiring dentists to take a full medical course is that, if one spends the time and energy to take a medical course, he is almost sure to practice medicine and not dentistry nor stomatology, for the reason that the returns in medicine, both intellectual and financial, are greater than they are in dentistry or stomatology.

To be a perfectly fitted stomatologist requires greater versatility and training than does either dentistry or medicine alone, for a stomatologist must be first a skilled mechanic and artist, then a well-posted physician and surgeon. I believe that neither the fees nor the breadth of the the scope in this specialty are great enough to attract many to it who have the capacity to do both well, and I imagine that for some time in the future, as in the past, we shall have many dentists and very few stomatologists. I believe that there should be a special degree conferred in certain colleges to distinguish a stomatologist from the average dentist.

Dr. Stewart L. McCurdy, Pittsburg, Pa.: As a practitioner of general surgery as well as surgical stomatology, I have observed first, the lack of knowledge on the part of the dentist of the general medical branches, and second, the lack of knowledge on the part of the medical practitioner of oral diseases.

The remedy, it appears to me, would be plain, i. e., more medicine for the stomatologist (dentist) and at least some oral pathology and surgery for the medical student. The physician knows practically nothing of the pathology of the mouth. That the future will have in store for students a four-year course for dentists and a five-year course for medicine is a probability. When this does occur the method of instruction will, no doubt, be for students in both medicine and dentistry, to take the same course for two years. This will leave two years for the stomatologist to complete his course and three years for the student who desires to specialize in medicine to complete his course.

Dr. J. P. Gray, Nashville, Tenn., Secretary of the Department of Dentistry, Vanderbilt University: I am not quite sure that I could recommend a full medical course for dental students. I fear the length of time would prevent a student from first taking a medical course and a dental, and that the majority of students applying for a dental course would not go further after taking the medical. Most men would not have the means to do this extra amount of work. I would require the dental student to take the first two years of the fundamental branches of medicine with the medical students, being required to do all the laboratory work required of the medical student; then I think it would be better--if it could be so arranged with the medical schools—to eliminate some parts of the medical course, and in addition to substitute some studies of the dental course. Some of the work in the dental schools might be eliminated, but most of it should be given in connection with the medical course. There is no reason why gynecology, obstetrics and other medical branches could not be taken out of the medical course for the dental student and yet a degree be given commensurate with the work done by the latter. The dental branches require considerable amount of technic—more than is required in ophthalmology and otology—and a student should have some of this technic in his junoir and senior years. By the elimination of the extra branches in medicine and the addition of the dental it seems that a sufficient amount of technic and dental instruction could be imparted, and a student would still have a course equal to that of some other branches of medicine. If we are to give a purely medical course, I should make the students take up the study of the fundamental branches the first two years with the medical students; then I should give the dental course with a few of the medical studies, leaving the technic for the third and fourth year of the school, and add one year more to the completion, making five years.

I have not found in my experience of twenty years as a teacher that medical men coming to us—and we have had quite a number— make first-class operators. It may be that we have received those who have been failures in medicine. I can recall but one man who made a good student and who really was highly benefited by having studied medicine. I think that the medical schools are making a mistake in not allowing some credit to students who have attended dental schools. I know the courses given here in the fundamental branches, such as anatomy, physiology, histology, pathology, materia medica and therapeutics, should give a man at least one year off, if not one and one-half years in the medical school. If they were not satisfied with them after an examination, they might then dismiss the student. The medical colleges could find out the dental schools worthy of recognition. I do not think the course in some dental schools worthy of recognition by the medical schools.

Dr. N. P. Colwell, Chicago, Secretary of the Council on Medical Education: No man should pose as a specialist in the treatment of any class of human ailments, whether diseases of the eye, of the stomach, of the nervous system, or of the mouth, unless he has first had a full medical, training. Whether every dentist should first have a complete medical training or not is a question which may need considerable discussion. It may certainly be said, however, that there should be some way to distinguish between the dentist who has had a complete medical training and the one who has not. Certain it is that the former is in position to recognize more clearly causes of certain diseases of the teeth and gums than the latter, and, therefore, is enabled to treat such conditions more intelligently.

Suppose, therefore, that all dentists shall first have a complete medical course, followed by the course in dentistry. This would mean, first, that all dentists, would have a better preliminary education which would include a training in physics, chemistry and biology; second, they would have more thorough courses in the fundamental medical sciences of anatomy, physiology and the like, which are now only briefly touched on in the usual course in dentistry; third, they would all have a knowledge of the etiology, symptoms, prognosis and treatment of the more common diseases not now possessed by most dentists; fourth, it would mean a higher order of intelligence of those who are to practice dentistry, and last but not least, it would mean more intelligent treatment of patients, more permanent fillings in tooth cavities, more prompt treatment of many diseases of the gums and the prevention of many such diseases and of their sequelae, such as diseases of the antrum of Highmore, of the frontal and accessory sinuses and the like. Furthermore, if all dentists were so educated there would doubtless be a more effective movement to educate the public to more proper care for the teeth.

The chief objection would be the more prolonged course of study required, but if the dental profession, like medicine, is already overcrowded, then the future should possibly develop fewer but better dentists, even as in medicine the demand is for fewer but better doctors, or, as President Faunce puts it, "fewer doctors but more doctor." After the completion of the four-year medical course it would not require an additional three years to complete the course in dentistry, since many of the subjects would already have been completed in the medical course and much more thoroughly studied than in the usual course of dentistry. Anatomy, physiology, chemistry, pathology and possibly over half of the dental course would have been completed. The time for the two courses might be further shortened by allowing the election of certain special courses in dental anatomy, dental physiology, dental pathology, dental surgery and the like, thereby leaving for a fifth year the strictly dental subjects and for clinical courses in the special study of diseases of the mouth. Since many systemic diseases have some of their first symptoms appear in the mouth which would be discovered by the routine examination of the gums and teeth, it is very important that the subject broached in Dr. Talbot's very able paper be given the most careful consideration.

Dr. Arthur D. Bevan, Chicago, Chairman of the Council on Medical Education: The problem of the stomatologist's education is certainly a difficult one to solve. It seemed to me that if five years could be devoted to the study of dentistry, the student might put in the first two years in the scientific laboratory branches with the regular medical student, and that he might take one year in general clinical medicine and surgery and two years limited to oral surgery and dentistry. With such a training, I should think that he should receive the same sort of a degree and be recognized as a doctor of medicine limiting his work to stomatology and with just as much right as the men who are limiting their work to diseases of the eye, ear, nose or throat It would seem to me that such a course would give him a broad training for his work.

Dr. John M. Dodson, Chicago, Dean of the Medical Courses, University of Chicago: True stomatology bears the same relation to general medicine and surgery that is borne by ophthalmology, laryngology, gynecology or any of the specialties which have to do with particular organs, or portions of the body. Doubtless dentistry, in its present narrow or restricted form as a mechanical art, has grown up independently of medicine because the purely mechanical side of that vocation—the filling of teeth, the manufacture of artificial substitutes for the teeth, etc.—occupies so large a part of the time of the dental practice.

It seems clear to me however, that the disease of the mouth and teeth are so intimately related to pathologic states in other portions of the body that they can be intelligently and successfully treated only by one who has been thoroughly educated in medcine. I believe, therefore, that the stomatologist of the future will be, first a well-educated man, who has secured as the very minimum of preparation for his professional study, the equivalent of four years in high school, plus two years in a literary or scientific college; and, second, an educated physician which means, at the present time, four years of training in a well-equipped medical school, this to be supplemented by at least a year of training, as an intern, in a general hospital or its equivalent in general practice outside a hospital and, third, a specialist, qualified in the treatment of diseases of the mouth and teeth by at least two sessions (eighteen months) of attendance and training in the dental department of a university. A similar course of training for the other special branches of medicine is now provided in at least one of the university medical schools.

Possibly the individual then adequately prepared for the practice of stomatology will find it of advantage to relegate a considerable portion of the purely mechanical part of his practice, *e. g.*, the manufacture of artficial dentures, of crown and bridges, of gold and porcelain inlays, to artisans who will devote themselves wholly to such work. It is idle to contend that because the fundamental medical sciences (anatomy, physiology, etc.) are included in the curriculum of the dental school, the dental student receives an education in these branches at all comparable to that acquired by the medical student. The medical school was forced to abandon the practice of granting to the dental graduate, advanced standing in these subjects because his preparation in them was found to be wholly inadequate for the intelligent study of clinical medicine. When dentistry or stomatology comes to be generally taught in a thorough and effective manner, then and then only will it be entitled to rank among the recognized special branches of scientific medicine. There are to-day a number of stomatologists of this type—men who are graduates in medicine as well as in dentistry.

Dr. Frederick B. Noyes, Chicago: The development of the last ten years has divided medical education into two periods of two years each, the first devoted to the study of the medical sciences, the last to the application of them to the practice of medicine. The stomatologist requires the same training in the medical sciences, but the second period should be given to their application in the treatment of diseases of the mouth. That this will be about the line of progress in the future seems certain to me. It is absurd to suppose that the stomatologist should have the same training in gynecology, obstetrics, amputations, abdominal surgery, or histology, pathology, materia medica, etc., as the medical men.

After fifteen years of teaching in the dental dchools, I feel that the greatest fault to be found, at least with the better schools, is the quality of the elementary work, and that this spoils the quality of all the professional studies. This condition is not confined to the theoretical or scientific subjects, but is true also of the technical and mechanical departments. There has been too much crowding for the accomplishment of a large amount of dummy dental operations requiring the construction of a specimen of every new type of bridge or denture, and especially every pet device of the instructor and too little attention to the acquirement of skill. Judged from the standpoint of the fine mechanic, the average dentist is not skilful. There has been too much tendency to despise the mechanical side of dentistry. I believe that at the present time the dental schools fail to make a reasonable percentage of their graduates mechanically skilful, and that those who excel in skill do so because of a training acquired outside of the dental schools.

It seems very desirable that the stomatologist's course should be so planned that it could be begun immediately after high-school graduation, but so as to induce as many men as possible to take more collegiate work before beginning their professional studies. This would be accomplished if two years of collegiate work before the first two years of professional work would give the B.A. or the B.S. degree. But students entering professional work direct would receive only the professional degree. The decision between stomatology and medicine should be made at the beginning of the professional work, or, in the former course general mechanical training should be given, with the fundamental medical science. This should be in the form of applied mechanics of the finest order and designed for the acquirement of skill in the handling of tools. In the last two years the technical training should be in the application of the skill acquired to the performance of dental operations and the pursuit of the medical sciences into the field of stomatology. Finally let me repeat that at the present time failure to do thorough work in the elementary subject, both scientific and practical, is the greatest fault. Three years is too short a time to do what is undertaken in the dental school and more important than this, dental students are not required to work as hard as students in other departments of the university. The work is arranged for the poorest students. The best students find it is easy to do what is required and have not sufficient incentive to do any more.

Dr. George V. I. Brown, Milwaukee, Wis., Professor of Oral Surgery and Oral Pathology in the State University of Iowa: The ideal medical course, one comprehending premedical education leading to a degree in arts or science, then four years of medicine, at least a year of hospital service, and then approximately two years of post-graduate preparation for special or other practice, and at best a few additional years before an independent practice can be acquired, is no longer ideal when one stops to realize the hardships it entails on the less fortunate though often worthy students and the crippling of the best usefulness of many other individuals by destroying the first enthusiasm of youth and postponing the day when they can seek independence, in this day of intensely active business and professional pressure, until they have arrived at a mature age, when such knowledge comes slowly, or at the expense of much hardship if at all.

All this does not imply that these courses should be shortened, for on the contrary they are essentially necessary, but it does mean that there must be reconstruction of the educational system from the primary school up, in order that more advanced or at least better work may be done before entrance to college. The gymnasium in Europe accomplishes this. Why not our high schools?

The first year's course as now arranged for dental students in university and the best other schools requires attendance on nearly all of the same classes as the medical students, and in addition thereto a considerable number of hours of special laboratory work. The result is, that students of dentistry cannot as a rule do the thorough work required of students of medicine, and to this extent at least, the educational result is defective; therefore these students are not entitled to full medical credit for this portion of the work. Slight rearrangement of the curriculum by having one freshman branch carried forward to the sophomore year and an equivalent reduction in the number of laboratory hours for operative or prosthetic technics, would enable then to fully qualify in all medical branches taken at this time and yet have the advantage of early manual training. Continuance of this system from year to year would create practically no confusion and in the fourth year which as now arranged in medical colleges is largely devoted to clinical work, the branches carried forward could be completed and dental operatory work begun. An additional year of clinical, surgical, medical and dental operatory work would in five years give better preparation than could be accomplished even in six years if either medicine or dentistry alone were completed and the other taken as a post-graduate course.

The plan of work outlined could, if desierd, be inaugurated at the beginning of any term in any high-grade medical college without serious disturbance of courses as now arranged, and is sufficiently elastic to keep pace with any changes in the curriculum that advancing conditions might make necessary without creating disharmony in the schools directly interested.

Dr. Fred. C. Zapffe, Chicago, Secretary of the Association of American Medical Colleges: I have the highest regard for dentistry, and I would not for a moment have it thought that I disparage the tremendous advances that have been made in that specialty. But dentistry has arrived at the parting of the ways. It must establish itself either as a profession or a trade, an art or a science. It has always seemed to me that dentistry, to be regarded as a profession, should ally itself with the professions. As it is to-day, dentistry is neither a profession nor a trade. It is practically in the same class as optometry. Dentistry has extended beyond its original scope—the treatment of diseases of the teeth; it now comprises the treatment of all the diseases, injuries and malformations of the mouth. It is really on a par with ophthalmology, otology, laryngology, etc. And yet, the ophthalmologist, before he can be regarded as proficient in his specialty, must have a medical foundation and from three to five years of special study. Medical educators are keenly alive to the necessity of devoting more time in the colleges course to general medical education, and less to the so-called specialties in medicine. The specialists insist on thorough preparation in the fundamental and subsequent study in the chosen specialty, because a thorough knowledge of the fundamentals in medicine is a prerequisite to competent and efficient specialization. But note that there must be the preliminary training in the fundamentals. At least half of the course in dentistry to-day is given over to the study of fundamentals, which are the same as those in medicine. Dentistry must properly be regarded as a part of medicine. It is based on a proper comprehension and understanding of the causes and the development of diseases of the mouth and teeth. It is no longer merly a matter of knowing how to prepare a cavity and to fill it with suitable material, or of knowing how to construct an artificial denture. That is the mechanics of dentistry and is on a par with the work of the optician.

The dentist must know why the diseased condition is present; what it means, and how it can best be treated. He must have more than finger training; he must know pathology. To know pathology, he must know anatomy, physiology and chemistry.

He can get such training in the medical school better than anywhere else. Why not, then, elevate the practice of dentistry to the distinctly professional plane? Why not establish it as representing special study? Why not make the dentist in every way a better man professionally and educationally than he is to-sday. Let him get his special training after he is well grounded in the fundamentals. The specialty of dentistry will then occupy the same plane as ophthalmology and the dentist will be a graduate in medicine, with special training in the treatment of diseases of the mouth—in other words, a stomatologist.

Just how much special training he should have the stomatologist must determine. Schools of stomatology and stomatologic societies will take care of that. An education in stomatology will not be any more costly than an education in dentistry, and the graduate from such a course will be better equipped, better prepared to practice his specialty and regarded as a professional man and not as a dentist.

Dr. H. A. Potts, Chicago: At the meeting of the National Association of Stomatology, held in Paris, in 1907, it was settled that a complete medical education must be required of every dentist. Granting this, the problem of securing it confronts us.

The dental school which requires a medical degree for entrance will go begging for students, and many who do enter will be those who have made a failure in medicine. On the other hand, the best-equipped dentist will be the one who, after receiving his dental degree, continues his medical studies, finally receiving his medical degree, followed by an internship in the free dental dispensary, much the same as is now required by some medical schools.

One solution of the problem would be, first, to have the dental colleges and medical colleges under university control, with the same requirements for admission to each; second, with the dental college and medical college, belonging to the same university, be situated near each other; third, that the departments of dental histology, dental physiology and dental pathology be established in the medical school; fourth, that the medical students and dental students take the same courses for the first two years; fifth, that the dental students then pursue their clinical dentistry and the medical students pursue their study to completion.

Thus the medical student will have gained more kEowledge of dental conditions as they are related to medicine. and the dental student will have acquired some knowledge of medicine, which is sadly lacking under the present regime.

The dental student, and many a graduate as well, looks on a patient with some pathologic condition of the mouth as being "in his line" or "out of his line" of treatment. When he has filled all the carious cavities, he thinks his work is done. He should be able to group the whole pathologic picture and know the relation of conditions in the mouth to other conditions of the body which require medical treatment, in order that he may intelligently call in cousel or refer the patient to someone capable of caring for him. In other words, he must be able at least to make a probable diagnosis of the case in general, just as in medicine, it is not enough that a physician make a diagnosis of pulmonary tuberculosis; he must be able to say how far advanecd it is; he must know the condition of the digestive and eliminative organs, what complications exist, whether the patient is a menace to the health of others, or not. Tersely, he must be able to make a complete diagnosis.

The dentist should have courses in physical diagnosis, both clinical and laboratory. His experience in handling patients in the free dispensary of the medical school will be of inestimable value to him in handling his own dental patients. Only by actual work in inspection, palpatio, percussion and ausculation on patients suffering from various diseases will he be able to grasp the whole picture and becomes familiar with the living pathology of the mouth, and see its general connection with general pathologic conditions. If he fails in this, his medical degree and the time taken to attain it have been for naught.

Dr. Eugene S. Talbot, Chicago: I am surprised and pleased at the unity of opinion expressed by the able teachers coming from different parts of the country and occupying such varied positions in the teaching world both in medicine and dentistry. On one point all agree and that is all dentists should be stomatologists practicing under a medical degree. All seem to agree that the medical course should be so arranged that the fundamentals of medicine should be the foundation on which to build all specialties. First obtain the medical degree and the specialties studied afterward. This will place all specialties on an equal footing. This method seems reasonable and easy to accomplish.

Like the medical schools in this country, there are more dental schools than can exist. There is no need of more than one school in a state. Let the weaker schools unite with the stronger or disband. About fifty-four medical schools have either given up or joined stronger and better equipped schools in the past five years. Twenty or twenty-five dental schools can go out of existence and never be missed. Let twenty-five of the best schools become actual departments of universities and an ideal specialty can thereby be developed.—*Journal A. M. A.* June 28, 1910.

